# Biological Activity and Chemical Constituents of Red and Brown Algae from the Persian Gulf 

**Published:** 2013

**Authors:** Amir Reza Jassbi, Maryam Mohabati, Saba Eslami, Jelveh Sohrabipour, Ramin Miri

**Affiliations:** a *Medicinal and Natural Products Chemistry Research Centre, Shiraz University of Medical Sciences, Shiraz, Iran.*; b *Institute of Ocean and Earth Science (IOES), Faculty of Science, University of Malaya. *

**Keywords:** *Hypnea flagelliformis*, *Cystoseira myrica*, *Sargassum boveanum*, Antimicrobial activity, Antioxidants activity

## Abstract

Different solvent extracts of a red algae, *Hypnea flagelliformis*, and two brown algae, *Cystoseira myrica *and *Sargassum boveanum*, which were collected from the Persian Gulf coast were subjected to different bioassays, including: 1,1-diphenyl-2-picrylhydrazyl (DPPH) free radical scavenging assay, antibacterial and antifungal activity by thin layer chromatography (TLC)-bioautography, agar disc diffusion (ADD) and nutrient-broth micro-dilution (NBMD) bioassays. The water extracts were found to have the most antioxidant activity. The antibacterial minimum inhibitory concentrations (MIC) of the active extracts were determined for the susceptible organisms, *Staphylococcus aurous *and *Bacillus subtilis, *using NBMD bioassays. The active substances were identified as free fatty acids (FFA), by using gas chromatography-mass spectrometry (GC-MS). After derivatization to their methyl esters, their concentrations were measured by using GC- lame ionization detection (GC-FID). In addition to the fatty acids, fucosterol, cholesterol and 22-dehydroxychlosterol were detected as the major sterols in *S. boveanum *extract using GC-MS analyses.

## Introduction

Recently the marine organisms from the Persian Gulf and Gulf of Oman have attracted the attention of several investigators from the surrounding countries including Iran (1-6). From about 150 species of the marine algae collected in the Iranian coast of the Persian Gulf, only a few have been subjected to biological and chemical investigation (7). 

Among the studied species, ethyl acetate and 50% methanol extracts of the red algae *Hypnea flagelliformis *showed significant toxicity for *Artemia salina *larvae in brine shrimp toxicity tests (8). In addition to the toxicity test, the above extracts were subjected to antibacterial and antifungal bioassays using different test microorganisms (8). Four known steroids, 22-dehydrocholesterol, cholesterol, cholesterol oleate, and (22*E*)-cholesta-5,22-dien-3*β*-ol- 7-one and oleic acid were isolated from *H. flagelliformis*, collected from the Iranian coasts of the Persian Gulf (9). The hexane layer of the methanol extract of *Cystoseira myrica *was tested against cancer cell lines (3) and its water extract showed antiviral activity against herpes simplex virus type 1 (6). 

Recently, hexane, chloroform, ethyl acetate, and 70% methanol extracts of *C. myrica *were analyzed for their antioxidant activities and total phenol contents using ferric reducing antioxidant power (FRAP) and Folin-Ciocalteau assays respectively (10). Among the tested extract the 70% methanol-water extract was found the most active antioxidant (50.95 ± 4.33 m mol Fe II /100 g dried algae), containing 10.08 mg gallic acid in 100 g dry algal material (10). Finally, *Sargassum boveanum *which was collected from near the Bushehr seashore, was investigated for its total phenol and radical scavenging activity (5).

Considering the biologically active natural products extracted from different species of the above brown and red algae, we have subjected the algal extracts of *C. myrica*, *S. boveanum and H. flagelliformis *to evaluate their antibacterial, antifungal and antioxidant potential. In this paper we report the antibacterial fatty acids in the algal extracts or TLC semi-purified fractions identified by GC-MS. In addition to antimicrobial activity, the antioxidant potential of the algal water-extracts was correlated to their total phenol or carbohydrate contents. We also characterized the major steroids of the algal extracts. 

## Experimental

*General*

Column and flash column chromatography were performed on Merck silica gel (70-230 and 230-400 mesh, respectively). Analytical TLC experiments were performed on Merck silica gel 60 F_254_. Preparative TLC was performed on Merck silica gel 60F_254_ self-prepared on glass plates. Folin-Ciocalteau reagent, BF_3_ solution, cholesterol, nutrient broth and oleic acid were from Merck, DPPH from Fluka, quercetin and chloramphenicol were from Sigma-Aldrich. Sabouraud dextrose (SBD) agar and nutrient agar were from Liofilchem and fluid SBD and the paper discs were from Himedia laboratories.

*Algal material, extraction and purification of compounds*

The algae were collected from Qeshm Island coastal area and identified by one of us, JS (7). After grinding, 250 g of the dried *C. myrica *were extracted using 1 L dichloromethane (DCM) for 48 h at room temperature. The DCM extract was evaporated under reduced pressure to yield 3.3 g dry syrup which was subjected to column chromatography over a silica gel column (100 g, Merck, 70-230 mesh). The column was eluted with pure hexane and then the polarity of the mobile phase was increased with DCM followed by methanol. The similar fractions eluted with a mixture of DCM and hexane (rich in DCM) were combined (0.8 g) and then subjected to flash column chromatography over silica gel column using different ratios of hexane and chloroform as the mobile phase. Three fatty acids (their structures were not determined due to low concentrations) and fucosterol (9 mg) were purified by flash column chromatography and preparative TLC on AgNO_3 _impregnated silica gel (5% w/w) using chloroform or 5 percent acetone in chloroform as the mobile phases (Figure S.1).

**Figure S. 1:**
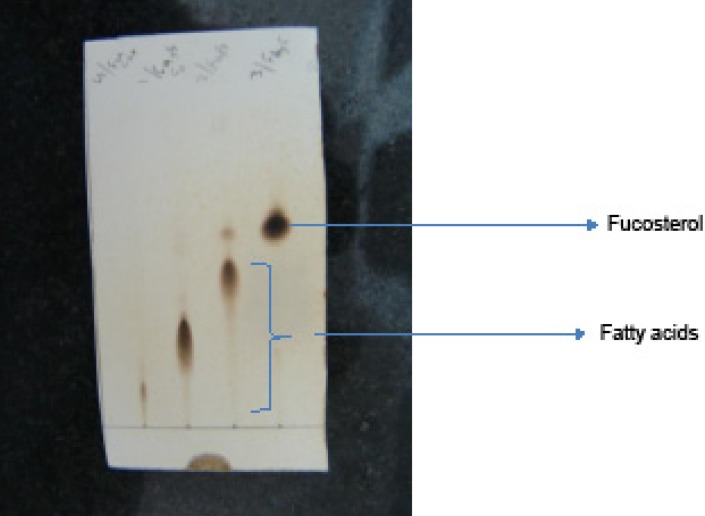
The fractions collected from flash column chromatography on Ag-Silica gel column and further purified by AgNO3 impregnated silica gel TLCs. The compounds corresponding to fatty acids were either degraded before GC-MS analyses or not sufficient to record the mass spectra

*Determination of the free radical scavenging activity of the algal extracts by spectrophotometeric methods*

The free radical scavenging activity of the algal extracts was measured by the method of Blois (11) with some modifications (12, 13) and compared to that for quercetin as a standard radical scavenger. Because of solubility problems, 2 mL of a 100 μM solution of DPPH in methanol were added to 100-400 μL water algal extracts (100 mg algae was extracted in 1 mL water for 24 h) and then the final volume was adjusted to 4 mL with water. After 30 min shaking of the solutions in the darkness, the absorptions of the DPPH solutions were measured at 517 nm. The percentage of the reduced DPPH was calculated by the following equation:

Percentage of DPPH reduction = ((A_0_ – A_1_)/ A_0_) x 100), that A_0_ is the absorbance of the control (2 mL DPPH solution + 2 mL water), and A_1 _is the absorbance in the presence of sampl. The IC_50s_ were calculated by linear regression equations of the DPPH inhibition percentage from different concentrations of the algal extracts , the standard antioxidants, using Microsoft Excel and Curve Expert statistical programs and expressed as: mg algae extracted with solvent/ 1 mL 0.5 × 10^-4^ M DPPH. 

*Determination of the total phenol content in the algal extracts*

The total phenol contents of the algal extracts were determined by the Folin-Ciocalteau method as described previously with some modifications (14, 15). Briefly, to a 40 μL solution of the algal extract was added 3.16 mL water and 200 μL Folin-Ciocalteau reagent.The mixture was shaken well. 600 μL of a 0.25% sodium carbonate was added to this solution after 8.5 min incubation at room temperature. The above solution was further incubated at RT for 2 h and its absorbance was measured at 765 nm against the blank. The concentrations of the total phenolics were measured against a series of gallic acid standard solutions and expressed as mg equivalent of gallic acid in 1 g dry algal material (mg EG/g AM) (16).

*Antibacterial and antifungal agar disc diffusion method*

To examine the antibacterial activity of the algal extracts, three gram-negative bacteria (*Escherichia coli*: PTCC1330*, Klebsiella pneumonia*: PTCC1053 and *Salmonella typhi*: PTCC1609) and three gram-positive bacteria (*Staphylococcus aureus*: PTCC1112, *Staphylococcus epidemidis*: PTCC1114, *Bacillus subtilis*: PTCC1023) were chosen and tested in agar disc diffusion (ADD) bioassays. The minimum inhibitory concentrations (MIC) of the active extracts were determined using nutrient broth micro-dilution (NBMD). For antifungal bioassays the extracts were tested against the growth of *Aspergillus niger*: PTCC5010 and *Candida albicans*: PTCC5027 in ADD bioassays. 

Bacteria were grown in nutrient broth (Merck) overnight at 37ºC. Before seeding the agar plates, their optical density were measured at 600 nm and adjusted to 0.1. The crude extracts (methanol, 80% methanol in water, dichloromethane and water) and the compounds separated from the preparative TLCs, were dissolved in the respective solvent and applied (5 mg) onto paper disc of 6 mm diameter. The dried papers were placed on agar seeded with 1 ml of the above bacteria suspension in a Petri dish. The Petri dishes were placed for 5 h at 4 °C that the metabolites could diffuse in the medium. The plates were incubated at 37°C for 18 h. The antibacterial activity was determined by measuring the diameters of the clean inhibitory zone (IZ) around each paper disc. Chloramphenicol was used as the positive control (17). The antifungal bioassay were performed with two test organisms *A. niger *and *C. albicans *with the procedure described previously (18). The microorganisms were grown in sabouraud dextrose broth (SDB) at 25 ºC for 48 h. sterile paper discs (charged with 10 mg algal extracts) placed on the agar media which was seeded with 1 mL fungi suspension in the SDB. The antifungal potential of the extracts measured as the clear IZ diameter around the paper discs after 48 h incubation at 25ºC and compared with standard chlotrimazol discs. 

*Antibacterial TLC bioautography*

Different algal crude extracts were analyzed by silica gel TLC plates (ethyl acetate-hexane=1:1 v/v, as the mobile phase) and the extracts were subjected to antibacterial TLC bioautography as described previously (17, 19). Briefly, a suspension of the gram positive bacteria *Staph. aureus*, *Staph. epidemidis *and *B. subtilis*, and the gram negative bacteria *E. coli *in nutrient broth were sprayed on the developed TLC plates. TLC plates were incubated for 4 hr at 37°C. The TLC plates were then sprayed with *p*-iodonitrotetrazolium violet (INT) solution (0.5 g/ 100 mL H_2_O) and incubated 1 h to visualize the purple color. White zones, representing the antibacterial constituents, were observed in case of *B. subtilis *(Figure S.2).

The corresponding bands in the preparative-TLC plates to the antibacterial zones on the TLC bioautography were divided into three parts from the baseline (Figure S.2). Each silica-gel band was extracted with chloroform and subjected to antimicrobial MIC bioassay and GC-MS analyses. Chloramphenicol was used as the standard antibacterial.

**Figure S. 2:**
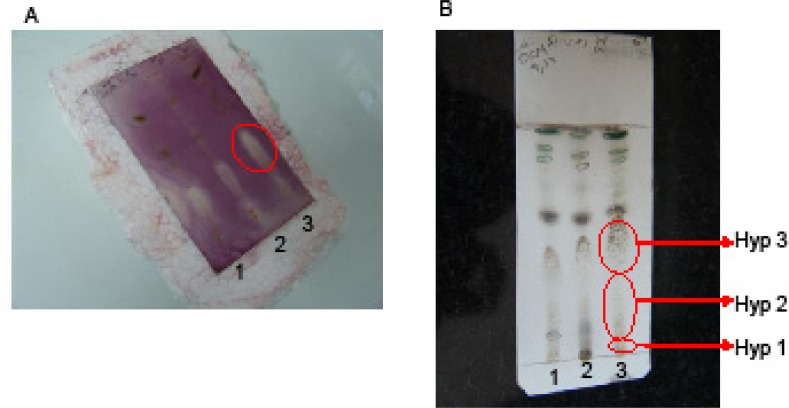
A) TLC bioautography on dichloromethane (DCM) extract of the algae (100 μg/spot; 1) *Sargassum boveanum *2) *Cystoseira myrica *and 3) *Hypnea flagelliformis*) on a silica gel plate, and the plate immersed in *Staphylococcus aureus *suspension and after incubation at 37 ºC sprayed with iodonitrotetrazolium chloride solution (INT). The pale zones indicated the presence of antibacterial compounds. B) Silica gel TLC using 5% acetone in chloroform as the mobile phase visualised by vanillin-sulphuric acid solution which was then used as a guide to separate the antibacterial constituents on the preparative-TLC in three bands for the algal extracts. Hyp stands for *Hypnea flagelliformis*

*Antibacterial minimum inhibitory concentration (MIC) using nutrient broth micro-dilution (NBMD)*

NBMD was performed by using serial two-fold dilution of the algal extracts added to bacterial suspension in nutrient broth as described previously (20, 21). The algal extract or positive control was dissolved in DMSO in different concentrations and was added (5 μL) to 95 μL of fresh media and 100 μL of bacterial suspension (OD=0.1 at 600 nm) in a 96-well microplate. The microplates were incubated at 37 ºC for 24 h in a shaking incubator and then 10 μL of 0.5% INT solution in water was added to each well and incubated for further 30 min at the above condition. The MIC was considered as the lowest concentration of the extract or antibacterial standard which discolored the purple color of the INT solution.

*Gas chromatography-mass spectrometry (GC-MS) and GC-flame ionization detector (FID)*

GC-MS analysis was carried out on an Agilent 7890A GC coupled to HP-6890 mass spectrometer operating in EI mode at 70 ev. The GC was equipped with a DB-5 MS (J & W Scientific column, 30 m X 0.25 mm i.d., 0.25 μm film thickness). To analyze the fatty acids, the oven temperature was programmed from 150 °C and after 4 min, rose to 250 °C at 4 °C/min and kept for 10 min at 250 °C. For steroidal analyses the oven temperature was set at 265 °C for 40 min. The carrier gas was helium (He) with a flow rate of 1 mL/min and the injector temperature was set at 260 °C in split mode (1:10). The injection volume was 0.1 μL for all of the samples. The GC-FID analyses were performed on the above instrument with the same analytical conditions, but the temperature of FID was set at 250 °C.

*Fatty acids methyl esterification*

The FFAs in the algal extracts were analyzed by GC-MS and after identification of the main FFAs in the crude extracts, they were transformed to their methyl ester derivatives using treatment of the crude algal extract with BF_3_ in MeOH (22). Briefly 250 μL 10% BF_3_ in methanol was added to 20 μg of the crude extract and heated on a boiling water bath for 1 h in a sealed glass vial. Three ml hexane and 1 mL water were added to the above solution, followed by extraction of the water layer with further 2 mL hexane after separation of the first hexane layer. The organic layer was dried over dry Na_2_SO_4_, evaporated under a nitrogen stream and dissolved in 1 mL hexane containing 0.1 mg thymol as internal standard and then subjected to GC-FID analyses.

*Identification of the constituents*

The free fatty acids or their methyl esters were identified by comparing their retention times, indices (23) and mass spectra recorded on GC-MS with those published in the *literature *(24, 25). The steroids were identified by comparison of their mass spectra and their retention times with those recorded for authentic sample.

## Results and Discussion

Dichloromethane (DCM), methanol (MeOH), ethyl acetate and water extracts of the algae *H. flagelliformis*, *C. myrica *and *S. boveanum *were subjected to the 1,1-diphenyl-2-picrylhydrazyl (DPPH) free radical scavenging assays. Water extracts showed the highest radical scavenging potential and among them *C. myrica *with IC_50_= 1.78 ± 0.96 mg algae extracted to scavenge 1 mL of a 0.5 × 10^-4^ M DPPH solution (mg AE mL^-1^ DPPH) was found to be more active (P < 0.0001) than *S. boveanum *(IC_50_= 7.73 ± 2.46 mg AE mL^-1^ DPPH) and *H. flagelliformis *(IC_50_= 8.75 ± 2.75 mg AE mL^-1^ DPPH) (Table 1). The water extracts of *C. myrica, S. boveanum *and *H. flagelliformis *contained 3.69 ± 0.12, 1.63 ± 0.13 and 1.44 ± 0.13 mg gallic acid equivalents in 1 g dry algal material (mg EG/g AM) respectively. The methanol extracts of the algae were several fold weaker in their radical scavenging potential compared to the water extracts. Unlike the water extract, the methanol extract of *C. myrica *showed the lowest radical scavenging activity (IC_50_=268 ± 33.57 mg AE mL^-1^ DPPH, p < 0.0001) and total phenol content (0.17 ± 0.03 mg EG/g AM, p < 0.0001) compared to those reported for *S. boveanum *(IC_50_=73.17 ± 4.53 mg AE mL^-1^ DPPH and 0.50 ± 0.03 mg EG/g AM) and *H. flagelliformis *(IC_50_=37.9 ± 9.71 mg AE mL^-1^ DPPH and 0.65 ± 0.05 mg EG/g AM). Quercetin was used as a standard antioxidant with IC_50_=1.79 ± 0.046 μg mL^-1^ DPPH.

**Table 1:** Total phenolic contents and DPPH radical scavenging potential of the methanol and water extracts of the algae

**Algae**	**Total Phenol **a **(methanol extract)**	**DPPH IC**_50_^b^ **(methanol extract) **	**Total Phenol ** **(water extract)**	**DPPH IC**_50_ **(water extract)**
*C. myrica*	0.17 ± 0.03 ^c^	268 ± 33.57 ^c^	3.69 ± 0.12 ^c ^	1.78 ± 0.96 ^c^
*S. boveanum *	0.50 ± 0.03	73.17 ± 4.53	1.63 ± 0.13	7.73 ± 2.46
*H. flagelliformis *	0.65 ± 0.05	37.9 ± 9.71	1.44 ± 0.13	8.75 ± 2.75
Quercetin	-	1.79 ± 0.046	-	-

^a^. Total phenol (mg eq. gallic acid in 1 g algae). ^b^. DPPH IC_50 _(mg algae extracted or μg quercetin/ 1 mL 0.5 × 10^-4^ M DPPH).^ c^. Significantly (p < 0.0001) different from those values reported for the other two organisms.

These data suggest that the water extract of *C. myrica *is a good candidate for isolation of marine antioxidants. The DCM and ethyl acetate extracts were not completely soluble in methanol and almost inactive in the above test conditions at the above concentrations. The co-occurrence of lower IC_50_ and higher total phenolic content also indicated that, as expected, the higher total phenolic content corresponds to better radical scavenging activity of the algal extracts. Pink spots observed on silica-gel TLC of the water extracts sprayed with 1% thymol in sulfuric acid-ethanol reagent (data are not shown) suggests that the carbohydrates or some glycosides are the major constituents of the water extracts of the algae. Since carrageenans, a kind of sulfated polysaccharide, previously reported in *H. flagelliformis*, and other polysaccharides such as laminaran, fucoidan and alginate which were extracted from different brown algae*, *are common water-soluble marine antioxidants, polysaccharides may be considered as potential active antioxidants in the water extracts of our studied algal extracts (26-28).

Among tested extracts for their antibacterial potential, the methanol extract of *H. flagelliformis *was active at 5 mg charged on the paper disc against *B. subtilis *and *Staph. aurous*, with 10 and 9 mm inhibition zones (IZ) on the agars. The MICs (10 mg AE/ml nutrient-broth: NB) were determined for both of the susceptible organisms (Table 2). The 80% methanol and water extracts of *S. boveanum *were active against *Staph. aureus *(MICs=10 mg AE/ml NB), while the DCM and methanol extracts of this algae were only active against the growth of *B. subtilis *(MICs=10 mg AE/ml NB)*. *The 80% methanol and methanol extracts of *C. myrica *showed antimicrobial activity against *B. subtilis *and *C. albicans *with IZ of 12 and 8 mm in the ADD tests, respectively. Chloramphenicol (10 μg/ paper disc) was used as standard antibiotic with 37 mm and 27 mm IZ for *Staph. aurous *and *B. subtilis*, respectively. Chlotrimazol (10 μg/ paper disc) was used as the positive antifungal standard and inhibited the growth of *C. albicans *(IZ=20 mm) and *A. niger *(IZ=25mm). The MICs (0.05 mg/mL) of chloramphenicol were higher for the resistance bacteria, *E. coli, K. pneumonia, S. typhi *and *Staph. epidemidis *(MIC = 0.025 mg/mL) compared to those obtained for the susceptible microorganisms, *B. subtilis *and *Staph. aureus *(MIC = 0.0125 mg/mL, Table 2). 

**Table 2:** Antimicrobial potential of different algal extracts (MeOH = methanol, DCM = dichloromethane) by agar disc diffusion and nutrient-broth micro-dilution bioassays

**algae (rows)** **microorganisms**	***H. flagelliformis *** **(MeOH)**	***S. boveanum *** **(water extract)**	***S. boveanum *** **(80% MeOH)**	***S. boveanum *** **(MeOH)**	***S. boveanum *** **(DCM)**	***C. myrica *** **(80% MeOH**(	***C. myrica *** **(MeOH)**	**Chloramphenicol **	**Chlotrimazol **
*Staph. Aureus*	9.0 ± 0.2 mm ^a^, 10 mg/mL	10 mg/mL^b^	7.2 ± 0.2 mm, 10 mg/mL	NA	NA	NA	NA	32 mm^c^, 0.0125 mg/mL	-
*Staph. epidemidis*	12 ± 0.0 mm	NA	NA	NA	NA	NA	NA	0.025 mg/mL	-
*B. subtilis*	10 ± 0.0 mm, 10 mg/mL	NA	NA	8.2 ± 0.3 mm, 10 mg/mL	11 ± 0.2 mm, 10mg/mL	12 ± 0.2 mm	NA	27 mm, 0.0125mg/mL	-
*E. coli*	NA	NA	NA	NA	NA	NA	NA	0.05 mg/mL	-
*K. pneumonia*	NA	NA	NA	NA	NA	NA	NA	0.05 mg/mL	-
*S. typhi*	NA	NA	NA	NA	NA	NA	NA	0.05 mg/mL	-
*A. niger*	NA	NA	NA	NA	NA	NA	NA	-	25 mm
*C. Albicans*	NA	NA	NA	NA	NA	NA	8±0.3 mm	-	20 mm

^a.^ 5 mg of the extracts charged on each paper-disc placed on agar, the inhibition clear zone diameter (millimetres) was the average of three different replicates.

^b.^ Minimum inhibitory concentration of the algal extract in the bacterial suspension in the nutrient broth media (mg/ mL).

^c. ^The standard antibiotics, 10 μg/ paper disc. NA= not active.

To identify the antibacterial constituents of the active algal extracts, they were subjected to TLC-bioautography at 100 μg extract/ spot charged on the TLC plates (see the experimental). The detected antimicrobial zones on the TLC bioautography-plate corresponded to the fatty acids zones on an analytical TLC plate which was developed under the same analytical conditions (Figure S. 2). The active constituents were semi-purified by using silica gel-preparative TLC, analyzed by GC-MS and found to contain substantial amounts of free fatty acids (Table 3). 

**Table 3:** Free fatty acids identified in the antibacterial fractions detected by TLC-bioautography, isolated by preparative TLC and characterized by GC-MS in the algal extracts

**Compound**	**Cyst.1 ** ^a^	**Cyst.2**	**Cyst.3**	**Hyp.1**	**Hyp.2**	**Hyp.3**	**Sarg.1**	**Sarg.2**	**Sarg.3**	**Oleic acid**	**Chloramphenicol**
Tetradecanoic acid	0.62 ^b^	0.92	0.72	4.95	9.10	4.75	1.19	7.74	0.97	-	-
9-z-octadecanic acid	1.40	4.10	8.63	1.05	0.36	6.95	0.93	7.47	12.24	-	-
Pentadecanoic acid	-	-	-	0.89	2.11	1.19	-	-	-	-	-
Hexadecanoic acid	6.04	9.33	12.92	20.51	32.85	38.21	8.13	31.80	22.75	-	-
Octadecanoic acid	-	-	1.09	1.47	2.22	2.71	0.24	2.80	0.62	-	-
Total FFA %	8.07	14.35	23.35	28.86	46.63	53.80	10.49	49.80	36.59	100	-
UFA/TFA % ^c^	17.3	28.5	29.9	3.6	0.77	12.9	8.86	15	33.45	100	-
MIC for *Staph. aurous *^d^	-	0.5	0.25	1	-	-	-	0.5	0.25	>10	0.0125
MIC for *B. Subtilis*	-	0.5	0.5	1	-	0.5	-	1	0.125	>10	0.0125

^a.^ The antibacterial zones on TLC bioautography chromatogram were divided in three bands (Figure S.1) on preparative TLC for each algal extract: Cyst. = *C. myrica*, Hyp. = *H. flagelliformis *and Sarg. = *S. boveanum*.

^b.^ The concentration of the compounds were determined by GC-MS and expressed as the percentage of the constituents in the TLC semi-purified fraction (mg substance/100 mg semi-purified compounds) using oleic acid as external standard.

^c.^ The ratio percentage of unsaturated fatty acids to the saturated FFA.

^d.^ Antibacterial MIC: mg of the algal constituents purified by preparative TLC inhibited 1 mL of the bacterial suspension in nutrient broth.

We identified hexadecanoic acid and 9-(*Z*)-octadecenoic acid as the major fatty acid constituents in three different bands extracted by chloroform from preparative silica-gel TLC plates (Table 3). The minor constituents which were identified in the fractions and obtained from the preparative TLCs, were tetradecanoic, pentadecanoic and octadecanoic acids in different concentrations. The fractions, semi-purified by preparative TLC, were active against *Staph. aurous *and *B. subtilis *at MICs between 0.125 to 2 mg/ mL NB. The FFA constituted 8.1 to 53.8% (w/w) of the dry weight of the extracted preparative-TLC fractions in the algal extracts (Table 3). The crude DCM algal extract which showed antimicrobial zones on TLC-bioautography was transformed to methyl ester derivatives for GC-FID quantification of the possible FFAs (Table 4). Hexadecanoic acid was measured as the most abundant fatty acid in all of the algae, constituting 165.7±23.7, 58.2±4.5 and 95.9±11.5 μg in 1 gram of *C. myrica, H. flagelliformis *and *S. boveanum *dry weights, respectively. 

**Table 4:** Fatty acid composition (μg/g algal dry weight) of the algal dichloromethane-extracts after methyl ester derivatization analyzed by GC-FID

**Compound**	**RRI ** ^a^	***S. boveanum ***	***C. myrica***	***H. flagelliformis ***
Heptadecane	1698	-	-	4.1 ± 0.63
Tetradecanoic acid methyl ester	1718	8.1 ± 0.7	20.3 ± 3.5	8.2 ± 0.9
9-(*Z*)-Hexadecenoic acid methyl ester	1900	4.0 ± 1.2	54.6 ± 36.3	-
Hexadecanoic acid methyl ester	1922	58.2 ± 4.5	165.7 ± 23.7	95.9 ± 11.5
9,12-(*Z,Z*)-octadecadienoic acid methyl ester	2094	-	5.8 ± 1.8	-
9-(*Z*)-octadecenoic acid methyl ester	2101	8.4 ± 2.4	30.1 ± 10.6	8.1 ± 1.2
9-(*E*)-octadecenoic acid methyl ester	2107	-	16.1 ± 2.0	7.9 ± 1.03
Octadecanoic acid methyl ester	2120	3.6 ± 0.7	11.9 ± 1.5	4.6 ± 0.3

a. Relative retention indices (RRI) were compared to those obtained in GC-MS for correlation of the peaks in the two chromatograms

FFAs from marine micro- and macro-algae were reported as antibacterial against several test organisms, especially the gram positive bacteria *Staph. aureus*, *Staph. epidemidis *and *B. subtilis *(29). The unsaturated fatty acids, 9-(*Z*)-hexadecenoic acid and 6,9,12-(*Z,Z,Z*)-hexadecatrienoic acid were reported to be active at micro-molar concentrations, against multidrug-resistance *Staph. aureus*. The later compound was additionally inhibited the growth of *Listonella anguillarum, *a gram-negative pathogenic marine organism (29). Lack of correlation between the total identified FFA percentage and the antibacterial potential of the separated fractions suggests the presence of other antibacterial constituents in addition to the above FFAs (Table 2-4). Hexadecanoic (palmitic) acid was reported as one of the major constituents of *S. muticum *and an antifouling agent with antibacterial at 44 μg mL^-1^ and growth inhibition activity against diatom, *Cylindrothea closterium *(EC_50_ = 45.5 μg mL^-1^) and the macro-algae *Ulva lacuta *spores (3 μg mL^-1^) at low concentrations (30).

Polyunsaturated fatty acids (PUFAs) from *Ulva fasciata *and other macro-algae had algicidal activity against the harmful microalgae responsible for the red tide blooms (31). Considering the allelopathic interaction between the macro- and micro-algae reported previously, and our chemical analyses of the algae from the Persian Gulf, we may consider them as candidates to control harmful algal blooms in the future (32, 33). The GC-MS analyses of the algal extracts resulted in identification of three major free steroids in the DCM extract of *S. boveanum*. Fucosterol, cholesterol and 22-dehydroxychlosterol were identified by comparison of their mass spectra to those recorded in the literature and for cholesterol with co-injection of an authentic sample (Figure S.3).

**Figure S. 3:**
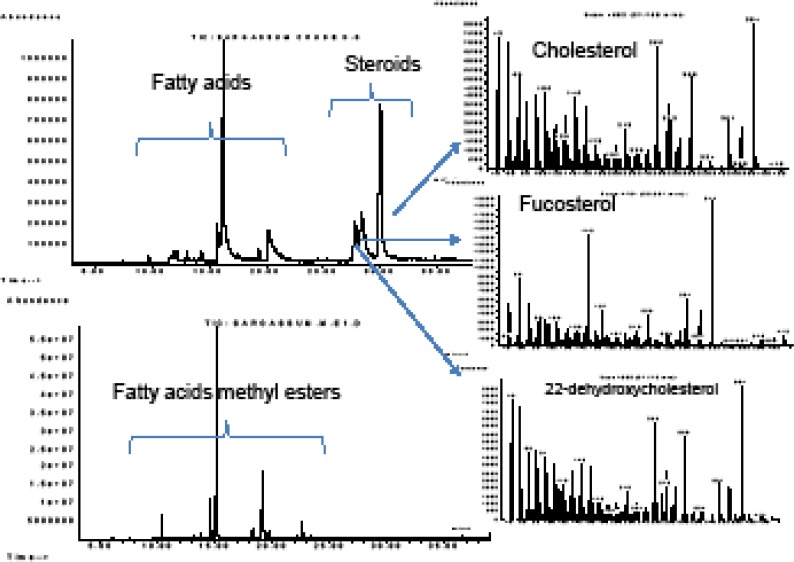
The GC-MS chromatograms of crude DCM extract of *Sargassum boveanum *before (upper) and after derivatization to methyl esters (lower chromatogram) and the mass spectra recorded for the free sterols
